# Catalase Protects Biofilm of *Staphylococcus aureus* against Daptomycin Activity

**DOI:** 10.3390/antibiotics10050511

**Published:** 2021-04-30

**Authors:** Cristina El Haj, Mads Lichtenberg, Karen Leth Nielsen, Thomas Bjarnsholt, Peter Østrup Jensen

**Affiliations:** 1 Costerton Biofilm Center, Department of Immunology and Microbiology, Faculty of Health Sciences, University of Copenhagen, DK-2200 Copenhagen, Denmark; celhaj6@gmail.com (C.E.H.); mlichtenberg@sund.ku.dk (M.L.); tbjarnsholt@sund.ku.dk (T.B.); 2 Department of Clinical Microbiology, Rigshospitalet, DK-2200 Copenhagen, Denmark; karen.leth.nielsen.01@regionh.dk; 3 Institute for Inflammation Research, Center for Rheumatology and Spine Diseases, Rigshospitalet, DK-2200 Copenhagen, Denmark

**Keywords:** biofilm, *Staphylococcus aureus*, daptomycin, catalase

## Abstract

Daptomycin is recommended for the treatment of *Staphylococcus aureus* infections due to its bactericidal activity. However, its mechanism of action is poorly understood. The involvement of reactive oxygen species (ROS) in the bactericidal activity of daptomycin has been proved against planktonic *S. aureus*, but not against the biofilm of *S. aureus*. Therefore, we evaluated if ROS contributes to the effect of daptomycin against biofilm of *S. aureus*. Biofilms of wild type, catalase deficient and daptomycin-resistant *S. aureus* strains were grown in microtiter-plates. After three days, the biofilms were exposed to daptomycin with or without thiourea in the presence of a ROS indicator. After overnight incubation, the amount of ROS and the percentage of surviving bacteria were determined. The bacterial survival was higher and the amount of ROS was lower in the wild type than in the catalase deficient biofilm, demonstrating a protective effect of catalase against daptomycin. The induction of cytotoxic ROS formation by daptomycin was verified by the addition of thiourea, which reduced the amount of ROS and protected the wild type biofilm against high concentrations of daptomycin. Accordingly, only the highest concentration of daptomycin reduced the bacterial survival and increased the ROS formation in the resistant biofilm. In conclusion, daptomycin induced the production of cytotoxic levels of endogenous ROS in *S. aureus* biofilm and the presence of catalase protected the biofilm against the lethality of the induced ROS.

## 1. Introduction

Biofilm-related infections have become a major clinical challenge. Bacterial biofilm-related infections exhibit tolerance to antibiotics and are protected against the host responses, making it extremely difficult to treat and eradicate infectious biofilm [1,2]. *Staphylococcus aureus* is one of the main microorganisms involved in biofilm-related human infections and its ability to develop antimicrobial resistance adds even more complexity to the treatment of these infections [3].

In recognition of these challenges, many new anti-biofilm therapeutic strategies are being investigated. The strategies are focussed on the eradication of biofilm-embedded bacteria, which present different metabolic conditions than those in the planktonic state of growth. In recent years, peptides have appeared as a novel approach to anti-biofilm treatment, as exemplified by using colistin to treat biofilms formed by Gram-negative bacilli. It has been suggested that colistin may be highly effective against dormant bacteria in anaerobic conditions, since colistin’s anti-biofilm activity is not dependent on the production of hydroxyl radicals [4].

Among the particular pipeline of anti-staphylococcal antibiotics, daptomycin has attracted attention due to its activity against growing and non-growing staphylococcal bacteria and daptomycin is effective against biofilm-related infections [5,6,7]. The mechanism of action of daptomycin is, however, not well understood [8,9], but the bactericidal effect of daptomycin against planktonic bacteria may rely on the endogenous formation of bactericidal reactive oxygen species (ROS) [10].

**Figure 1:**
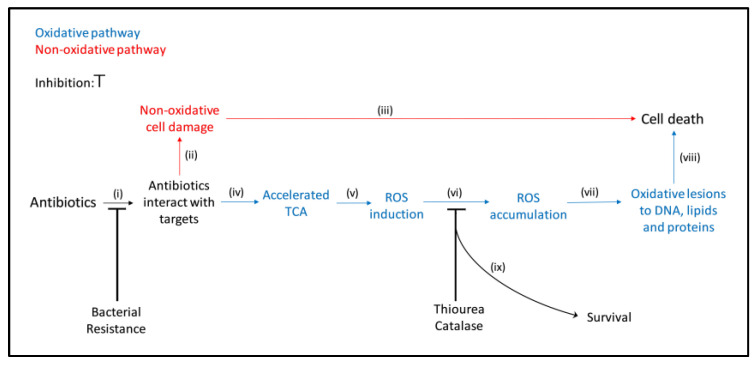
Schematic mechanisms of the main steps involved in the bactericidal activity of antibiotics. When antibiotics interact with their targets, (i) primary damage may occur. If this primary damage is severe and independent of oxidative stress, (ii) bacteria are not able to repair the damage (iii) and die (red). If the primary damage is insufficient to the kill the bacteria directly, the tricarboxylic acid cycle (TCA) (iv) may be accelerated, leading to the formation of ROS (v). Accumulation of ROS (vi) may cause secondary damage by introducing oxidative lesions in DNA, lipids and proteins (vii), resulting in killing of the bacteria (viii). Modified from Kohanski et al., 2007, Zhao et al., 2015, Van Acker and Coenye, 2017 [11,12,13]. In this study, the involvement of the oxidative pathway in the bactericidal activity of daptomycin was evaluated by the increased survival (ix) and the decreased ROS formation when ROS was removed with catalase or scavenged with thiourea (vi) and by preventing interactions with the targets in a resistant isolate (i).

With this in mind, we examined if daptomycin induces the formation of toxic ROS in *S. aureus* biofilm by estimating the effect of catalase and the effect of ROS scavenging by thiourea. The rationale for our experimental approach is outlined in Figure 1.

## 2. Results

### 2.1. Catalase Protects S. aureus Biofilm against Daptomycin

**Figure 2:**
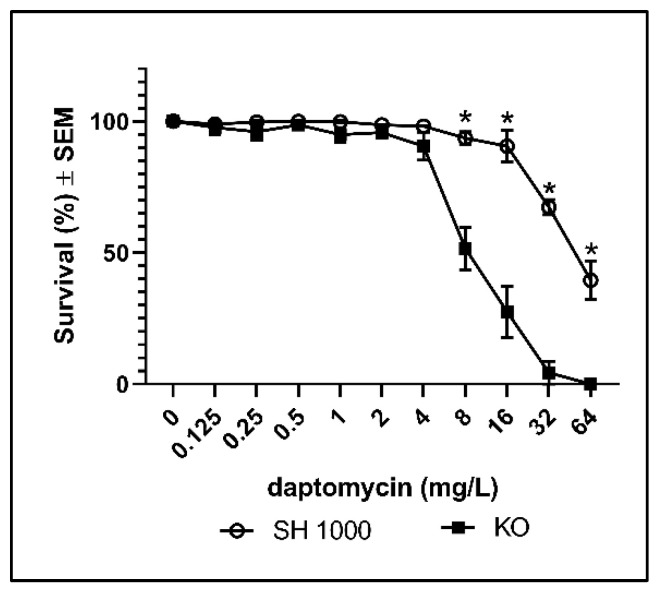
Percentages surviving cells demonstrating the protective effect of catalase against daptomycin treatment of *S. aureus* biofilms. Biofilms of the wild type (SH1000) and of the catalase deficient strain (KO) were grown for 3 days before overnight treatment with daptomycin. Mean ± SEM of triplicate experimental set-ups are shown. Statistical significance was determined using two-way ANOVA, followed by Bonferroni’s multiple comparison tests. * *p* < 0.05.

The contribution of ROS to the bactericidal activity of antibiotics is controversial [11,14,15,16], but examining susceptibility in mutants with deficient anti-oxidative mechanisms has long been recognized as a valid approach to estimate cytotoxic contributions of ROS to the activity of antibiotics [17]. Therefore, we compared the effect of catalase on daptomycin treatment using the wild type *S. aureus* strain (SH1000) and a *S. aureus* strain with deficient catalase activity (KO). We found that catalase deficiency was associated with significantly decreased survival during daptomycin treatment, demonstrating that catalase protects biofilm of *S. aureus* against daptomycin (Figure 2). The deficient catalase activity of KO was verified by the increased susceptibility to hydrogen peroxidase (H_2_O_2_) (Figure S1). A further indication of the involvement of ROS in the bactericidal activity of daptomycin on biofilm was demonstrated by the indicator of ROS, 2′,7′-dicholorodihydrofluorescein diacetate (DCHF), which was only enhanced significantly at daptomycin concentrations that significantly reduced survival in SH1000 and in KO biofilm (Figure S2). Altogether, these results indicate that catalase protects *S. aureus* biofilm against cytotoxic ROS induced by daptomycin.

### 2.2. Thiourea Protects S. aureus Biofilm against Daptomycin

**Figure 3:**
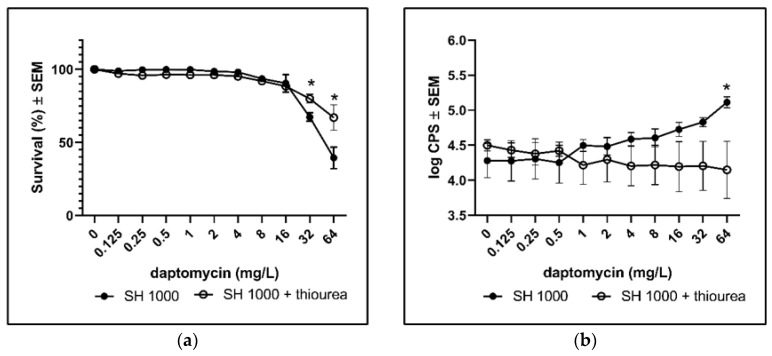
The rescuing effect of scavenging ROS with thiourea. Biofilms of the wild type (SH1000) were grown for 3 days before assessing the effect of the addition of thiourea during overnight treatment with daptomycin. (**a**) Bacterial survival was estimated by plating and counting of CFUs. (**b**) ROS formation according to DCHF fluorescence. Mean ± SEM of triplicate experimental set-ups are shown. Statistical significance was determined using two-way ANOVA, followed by Bonferroni’s multiple comparison tests. * *p* < 0.05.

To validate the contribution of ROS to the bactericidal activity of daptomycin, we added thiourea during treatment with daptomycin. Thiourea is a specific scavenger of hydroxyl radicals [18], which is a ROS that may contribute to the bactericidal activity against planktonic and biofilm bacteria [11,19]. The addition of thiourea to SH1000 biofilm significantly increased survival during daptomycin treatment (Figure 3a) and the DCHF fluorescence was significantly reduced (Figure 3b). Thus, the addition of thiourea further demonstrated that ROS might contribute to the activity of daptomycin on *S. aureus* biofilm.

### 2.3. Reduced Survival and Increased ROS Accumulation in Biofilm of a Resistant Clinical Isolate Requires High Concentrations of Daptomycin

**Figure 4:**
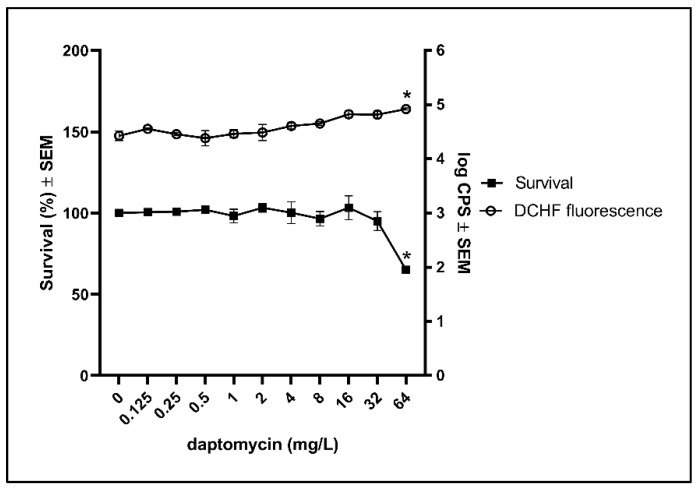
Effect of daptomycin on bacterial survival and the DCHF fluorescence in biofilm of a resistant clinical isolate. Biofilms of resistant clinical isolate (HUB512) were grown for 3 days before estimating the effect of daptomycin on the survival and ROS formation according to DCHF. Mean ± SEM of triplicate experimental set-ups are shown. Statistical significance was determined using one-way ANOVA, followed by Bonferroni’s multiple comparison tests. * *p* < 0.05.

To examine if biofilm of resistant clinical isolates may avoid the induction of cytotoxic ROS by daptomycin, as part of their protective strategy, we exposed biofilm of a resistant clinical isolate, HUB512, to daptomycin. Daptomycin was only able to reduce the survival and increase the DCHF fluorescence significantly at the highest concentration (64 mg/L) (Figure 4). This indicates that increased tolerance against daptomycin in *S. aureus* biofilm is associated with a decreased amount of ROS induction. Whole-genome sequencing data revealed a mutation, T345I, in *mprF*, which has previously been identified as a cause of lower susceptibility towards daptomycin [20,21].

## 3. Discussion

In this study, catalase and thiourea were able to protect three-day-old biofilm of *S. aureus* against daptomycin, indicating that ROS may contribute to the bactericidal activity of daptomycin on *S. aureus* biofilm. ROS may be involved in the bactericidal activity of aminoglycosides, fluoroquinolones and beta-lactams against planktonic bacteria [12]. In biofilm-embedded bacteria, a contribution of ROS to the antimicrobial activity of ciprofloxacin was demonstrated in biofilms of *Pseudomonas aeruginosa* [19]. However, the bactericidal activity of antimicrobial peptides may not depend on endogenous ROS, as in the case of colistin acting on planktonic cultures and the biofilm of *P. aeruginosa* [4,16].

The lipopeptide daptomycin has bactericidal activity against *S. aureus* in log and the stationary-phase of growth and has anti-biofilm activity against staphylococcal biofilm [22]. Daptomycin acts by disrupting cell membranes and killing by daptomycin does not require cell division or active metabolism. Daptomycin binds to calcium and integrates into the cytoplasmatic membrane where daptomycin forms ion-permeable transmembrane channels, causing changes in the membrane potential leading to cell rupture [23]. In contrast to other anti-staphylococcal antibiotics, daptomycin rapidly penetrates staphylococcal biofilms [6]. In addition, the frequency of development of daptomycin resistance is low, though strains with reduced susceptibility to daptomycin have been found in clinical practice [5]. Thus, current recommendations for the treatment of difficult infections encourage the use of daptomycin at high concentrations (8–10 mg/kg/day), in combination with other antibiotics [24].

Since daptomycin is a peptide-like antibiotic, it might be expected that the bactericidal effect of daptomycin resembles the mechanism of action of colistin on *P. aeruginosa*, which does not require endogenous ROS [4,16]. Nevertheless, Liu et al. [10] demonstrated a correlation between the activity of daptomycin and the formation of ROS in planktonic *S. aureus*. However, the contribution of ROS to the bactericidal effect of daptomycin has not been studied before against staphylococcal biofilm.

Compared to the wild type strain, the catalase deficient strain showed lower survival when treated with daptomycin, indicating that the wild type strain is more efficiently protected against endogenous H_2_O_2_. According to Kohanski et al. [11], H_2_O_2_ is a precursor for hydroxyl radicals, which are bactericidal and can be neutralized by the specific scavenger thiourea [18,25]. Therefore, the ability of thiourea to reduce the ROS indicator and increase the survival in the wild type biofilm treated with daptomycin is further confirming a contribution of ROS to the bactericidal effect of daptomycin on *S. aureus* biofilm. It may be argued that the protection of thiourea is derived from the induction of slow growth [17]. In our study, however, we treated it with 100 mM of thiourea, which did not affect growth (Supplementary Figure S3).

Estimation of ROS formation in biofilm may be achieved by using DCHF as an indicator for ROS [19]. In addition, treatments with concentrations of daptomycin resulting in decreased survival were accompanied with increased DCHF fluorescence in the wild type and in the catalase deficient mutant, which further emphasizes the contribution of endogenous ROS to the bactericidal activity of daptomycin on *S. aureus* biofilm. The decreased DCHF fluorescence during the rescuing of the wild type from daptomycin with thiourea also supports that endogenous ROS contributes to the bactericidal activity of daptomycin.

To further verify that accumulation of cytotoxic ROS is related to interactions between antibiotics and their targets, we treated a resistant clinical isolate with daptomycin. As expected, more daptomycin (64 mg/L) was needed to detect a significant antimicrobial effect on biofilm of the resistant isolate compared to the wildtype (32 mg/L). Accordingly, the DCHF fluorescence indicates the accumulation ROS in the resistant isolate was only increased at the bactericidal concentration of daptomycin. Since the resistant clinical isolate was more susceptible to H_2_O_2_ than the wild type (Figure S1), our results suggest the protective mechanisms of the resistant clinical isolate does not include enhanced anti-oxidative defence. Instead, we speculate, that the resistant clinical isolate disables the possibility of daptomycin to interact with its targets, which may lead to ROS formation [11]. The amino acid substitution in the *mprF* gene that we found in the HUB512 strain has previously been demonstrated in other isolates [20,21]. The mutations in the *mprF* gene result in increased net positive charge in the bacterial cell surface causing the repulsion of the positively charged daptomycin molecules [20].

Our results suggest that the stress on the membrane caused by daptomycin in *S. aureus* biofilm triggers the generation of cytotoxic ROS, as previously proposed [11]. Accordingly, interaction of daptomycin with its targets induces an increased activity of the TCA leading to super-oxidation of NADH by the enhancement of the electron transport chain, which results in superoxide formation. Subsequent dismutation of superoxide results in the production of H_2_O_2_, which may react with Fe^2+^ by Fenton reactions to produce hydroxyl radicals that is cytotoxic to *S. aureus* [25]. 

## 4. Conclusions

In conclusion, daptomycin activity against *S. aureus* biofilm depends on induction of ROS formation, as evidenced by the protection of the *S. aureus* biofilm against lethal oxidative stress provided by the presence of catalase and thiourea. Further studies are warranted in order to obtain a comprehensive in-depth identification of the species of ROS induced by daptomycin and the anti-oxidative mechanisms involved in the protection of *S. aureus* biofilm.

## 5. Materials and Methods

### 5.1. Strains and Susceptibility Studies

In this study, we employed the SH1000 wild type, the dual katA/ahpC knockout SH1000 *S. aureus* strains (KO) from University of Sheffield [26] and a daptomycin resistant *S. aureus* isolate (HUB512) from an osteoarticular infection at Bellvitge University Hospital (Barcelona, Spain). The dual katA/ahpC mutations disables the capacity of the strain to scavenge exogenous or endogenously produced H_2_O_2_ [26,27].

Minimal inhibitory concentrations (MIC) for daptomycin (Cubicin, Barcelona, Spain) were determined following standard recommendations [28] to classify the strains as susceptible or resistant to daptomycin according to EUCAST breakpoints and 50 mg/L of calcium were added to media. MIC for daptomycin was 0.5 mg/L for the wild type, 1 mg/L for KO and 4 mg/L for the HUB512 strain.

To verify the deficient catalase activity of the KO strain, the susceptibility of each strain to H_2_O_2_ was determined. Inoculums from overnight culture were adjusted to 10^6^ CFU/mL in Mueller Hinton Broth (Becton Dickinson, Kongens Lyngby, Denmark) in a 96 well plate, exposed to H_2_O_2_ at concentrations of 0, 10, 15, 20, 25, 30 and 60 mM of H_2_O_2_ and incubated in the dark at 37 °C for 1 h. Bacterial counts were determined before and after exposure by serial dilution in saline and plated on trypticase soy agar with 5% sheep blood (Becton Dickinson, Kongens Lyngby, Denmark) (Figure S1).

To determine the highest concentration of thiourea that does not inhibit growth, an inoculum of 10^6^ CFU/mL of each strain was exposed to 0, 50, 100, 150 and 200 mM of thiourea (Sigma, T8656) and incubated at 37 °C overnight. The growth was estimated by the optical density at 600 nm (Figure S3). Accordingly, it was decided to treat the biofilm with 100 mM thiourea.

### 5.2. Studies of Survival and Reactive Oxygen Species

The methodology used was a modification of a previously described method [19]. Briefly, an overnight culture of each strain was resuspended in Mueller Hinton Broth supplemented with 0.5% of glucose (*w*/*v*) (MHBG) to obtain an inoculum of 10^6^ CFU/mL. The final inoculum was added to each well of black microtiter plates with transparent flat bottom (Thermos Fisher scientific, New York, NY, USA) and incubated at 37 °C. After 3 days, the supernatant was replaced by 200 µL of daptomycin solution in MHBG in twofold dilution concentrations from 0 to 64 mg/L and 50 mg/L of calcium (with or without 100 mM of thiourea). To detect a broad range of ROS, 2′,7′-dicholorodihydrofluorescein diacetate (DCHF-DA; Sigma, Copenhagen, Denmark) was added to each well at a final concentration of 5 µM. Then, the plates were incubated in the dark at 37 °C overnight.

After incubation, the fluorescence of the biofilms was recorded at excitation/emission wavelength 485/535 nm on a plate-reader (Wallac 1420, Victor X2, Perkin Elmer, Boston, MA, USA). To eliminate the influence of autofluorescence caused by daptomycin, the fluorescence from treated cultures without DCHF-DA was subtracted. Surviving bacteria were determined by bacterial counts with a limit of detection of 10 CFU/mL.

All experiments were performed as biological triplicates.

### 5.3. Whole-Genome Sequencing

DNA of clinical isolate HUB512 was extracted using DNeasy blood and Tissue kit (Qiagen, Hilden, Germany), and libraries were created with Nextera XT (Illumina, San Diego, CA, USA). The isolate was sequenced on a NextSeq (Illumina) using paired-end technology (2 × 250 bp). Single nucleotide polymorphisms were identified using BacDist with *S. aureus* GCF_000013425.1 [29]. Analyses of BacDist output was performed in order to identify known polymorphisms in relevant genes causing daptomycin resistance (based on The Comprehensive Antibiotic Resistance Database (https://card.mcmaster.ca, accessed on 31 October 2020)).

### 5.4. Statistical Analyses

Data were analysed using Prism 8.0 (GraphPad Software Inc., La Jolla, CA, USA). All bacterial counts were represented as a percentage of surviving cells (mean ± SEM), taking bacterial counts from untreated control as a percentage equivalent to 100%. Comparisons of strains and effects of the addition of thiourea were performed by two-way ANOVA. The effects of daptomycin treatment on the percentage of surviving cells and the fluorescence of DCHF of each strain were evaluated by one-way analysis of variance (ANOVA). Adjustment for multiple comparisons was done by Bonferroni’s multiple comparison correction. Differences were considered statistically significant at *p* < 0.05.

## Data Availability

The data that support the findings of this study are available on request from the corresponding author. The data are not publicly available due to privacy or ethical restrictions. The raw reads from the sequencing are deposited in SRA under accession PRJNA725634.

## Supplementary Materials

The following are available online at https://www.mdpi.com/article/10.3390/antibiotics10050511/s1, Figure S1: Susceptibility of SH1000, catalase deficient and daptomycin resistant *S. aureus* strains to hydrogen peroxide, Figure S2: Survival and DCHF fluorescence in biofilms of SH1000 (a) and KO (b) treated with daptomycin, Figure S3: Percentage of bacterial survival with thiourea.
