# Supplementary material for: Catalase Protects Biofilm of Staphylococcus aureus against Daptomycin Activity
Source: Antibiotics (Basel). 2021 Apr 30;10(5):511. doi: 10.3390/antibiotics10050511 (PMC8146090; doi:10.3390/antibiotics10050511)
Supplement: Supplementary file 1 [file antibiotics-10-00511-s001.zip › antibiotics-1168778-supplementary.pdf]

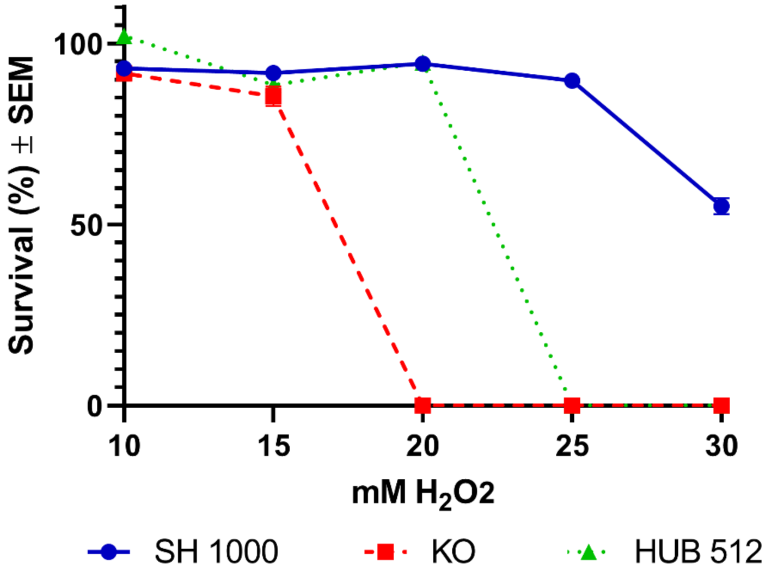

Figure S1. Susceptibility to hydrogen peroxide of the SH1000 strain, the catalase deficient strain and the daptomycin resistant clinically isolated *S. aureus* strain. The catalase deficient strain (KO) and the daptomycin-resistant strain (HUB512) were more susceptible to H_2O_2 peroxide than the wild type strain (SH1000), presenting bacterial counts under the limit of detection after exposure to H_2O_2 at concentration of 20 and 25mM, respectively.

a

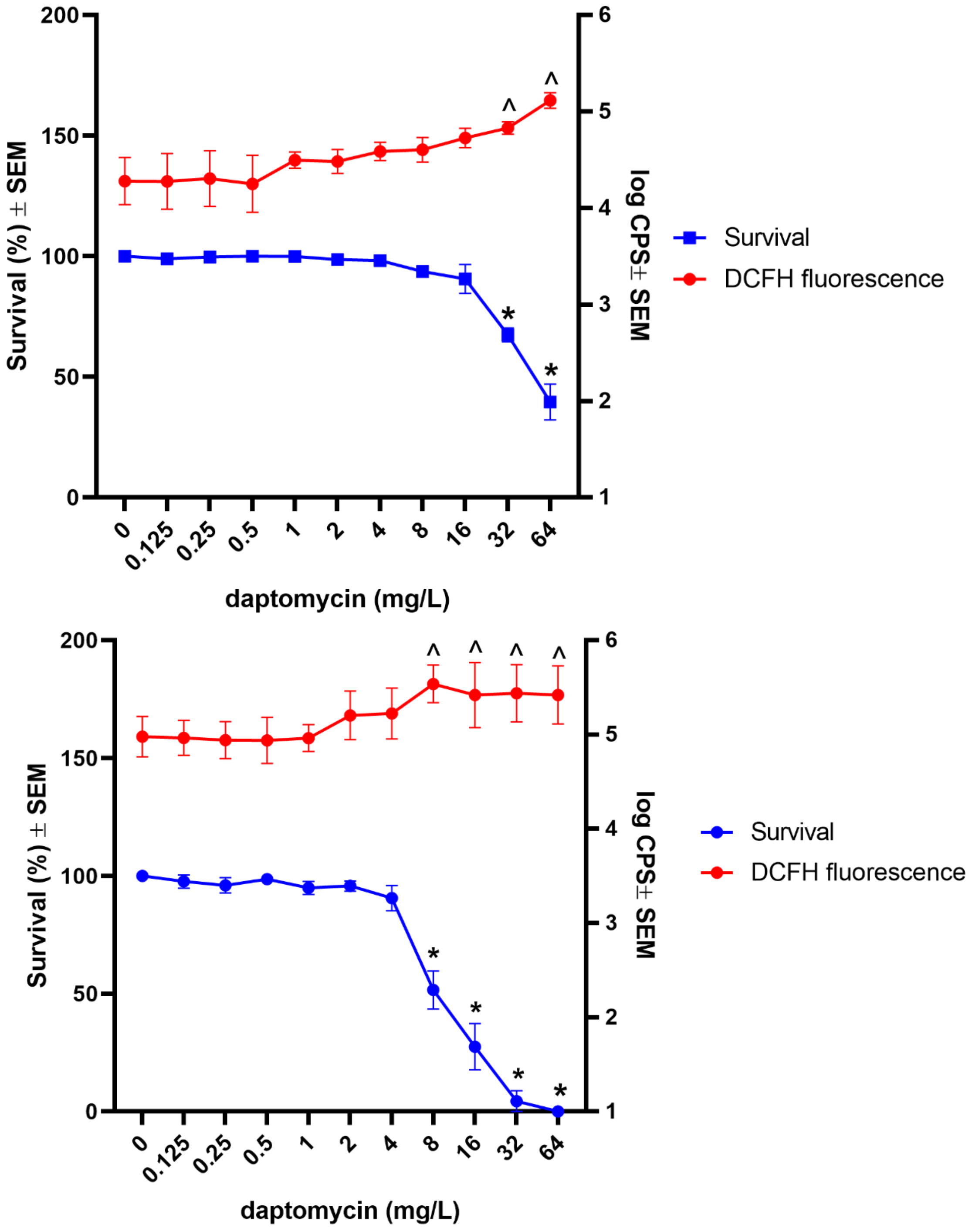

b

Figure S2. Percentage of survival and DCFH fluorescence in biofilm growing bacteria of SH1000 (a) and KO biofilm (b). *P < 0.05 vs survival bacteria (%) without daptomycin; ^P < 0.05 vs DCFH signal without daptomycin.

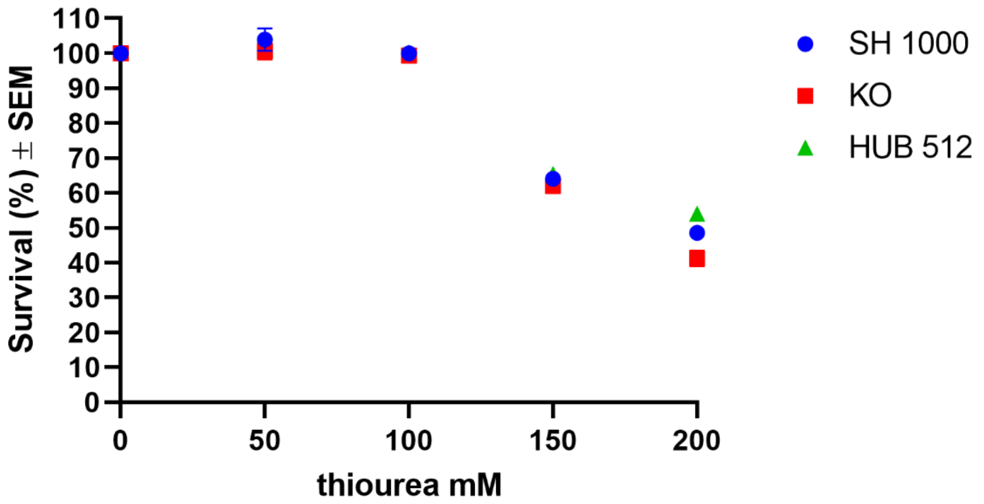

Figure S3. Percentage of surviving cells growing with thiourea
